# Inhibitory effect of Allium sativum and Zingiber officinale extracts on clinically important drug resistant pathogenic bacteria

**DOI:** 10.1186/1476-0711-11-8

**Published:** 2012-04-27

**Authors:** Iram Gull, Mariam Saeed, Halima Shaukat, Shahbaz M Aslam, Zahoor Qadir Samra, Amin M Athar

**Affiliations:** 1 Institute of Biochemistry and Biotechnology, University of the Punjab, Quaid-i-Azam Campus, Lahore, Pakistan

**Keywords:** Garlic, Ginger, Antibacterial activity, Extracts

## Abstract

**Background:**

Herbs and spices are very important and useful as therapeutic agent against many pathological infections. Increasing multidrug resistance of pathogens forces to find alternative compounds for treatment of infectious diseases.

**Methods:**

In the present study the antimicrobial potency of garlic and ginger has been investigated against eight local clinical bacterial isolates. Three types of extracts of each garlic and ginger including aqueous extract, methanol extract and ethanol extract had been assayed separately against drug resistant *Escherichia coli, Pseudomonas aeruginosa, Bacillus subtilis, Staphylococcus aureus, Klebsiella pneumoniae, Shigella sonnei, Staphylococcusepidermidis* and *Salmonella typhi.* The antibacterial activity was determined by disc diffusion method.

**Results:**

All tested bacterial strains were most susceptible to the garlic aqueous extract and showed poor susceptibility to the ginger aqueous extract. The (minimum inhibitory concentration) MIC of different bacterial species varied from 0.05 mg/ml to 1.0 mg/ml.

**Conclusion:**

In the light of several socioeconomic factors of Pakistan mainly poverty and poor hygienic condition, present study encourages the use of spices as alternative or supplementary medicine to reduce the burden of high cost, side effects and progressively increasing drug resistance of pathogens.

## Introduction

Microbial pathogenecity and other infectious diseases have been controlled by use of commercially available antimicrobial drugs since last many years. Tremendous use of antibiotics has developed multiple drug resistance (MDR) in many bacterial pathogens. The increasing drug resistance is the main hindrance in successful treatment of infectious diseases and to the control of microbial pathogenecity [1]. Similarly, preservatives like sulfites, nitrates, nitrites and antibiotics, are harmful for human health and have many side effects including headache, nausea, weakness, mental retardation, seizures, cancer and anorexia [2]. Development of drug resistance in pathogens and increasing interest of consumers for safe food forces to explore new antimicrobial agents [3]. Natural products are a major source of new natural drugs and their use as an alternative medicine for treatment of various diseases has been increased in the last few decades [4,5]. In comparison to the formulated drugs the herbs and spices have fewer side effects. They are also inexpensive, show better patient tolerance and are readily available for low socioeconomic populatation [6]. In recent years, in view of their beneficial effects, use of spices or herbs is gradually increasing not only in developing countries but also in developed countries [7].

The antimicrobial activity of spices is due to specific phytochemicals or essential oils [8]. The main factors that determine the antimicrobial activity are the type and composition of the spice, amount used, type of microorganism, composition of the food, pH value and temperature of the environment [9]. Several reports had been published that describe the antibacterial and antifungal properties of different herbs and spices. However, still there is little information about the exact mechanism of their antimicrobial action [10-16].

In the present study, *in vitro* antimicrobial activity of some local spices of Pakistan, that are routinely used in food, has been investigated against clinically important bacterial pathogens.

## Materials and methods

### Sample collection

Garlic (*Allium sativum*) and ginger (*Zingiber officinale*) used in the present study were purchased from the local market of Lahore, Pakistan.

### Bacterial strains

Eight different characterized drug resistant bacterial strains including *S. typhi, Shigella, P. aeruginosa, E. coli, B. subtillus, S. aureus, S. epidermidis* and *K. pneumoniae* were obtained from Sheikh Zayed hospital and Jinnah hospital, Lahore, Pakistan. The strains were maintained on Nutrient agar slants.

### Preparation of extracts

Three types of extracts such as aqueous, ethanol and methanol extract from each garlic and ginger were prepared separately. The fresh garlic cloves and ginger rhizomes were washed, peeled, sliced and sun dried for seven days. After drying, garlic and ginger slices were ground to fine powder separately using electric blender. 10 g powder of each garlic and ginger was soaked in 100 ml of distilled water, ethanol and methanol separately. The flasks were incubated at room temperature for 72 hours with shaking at 120 rpm. The crude extracts were centrifuged at 3000 rpm for 10 minutes at 25°C. The methanol and ethanol extracts were evaporated at 50°C while the aqueous extracts were evaporated at 80°C in rotary evaporator. All dried extract samples were dissolved in distilled water separately to the final concentration of 100 mg/ml and centrifuged again at 10,000 rpm to remove the undissolved residues. The extract solutions were stored at 4°C. Garlic aqueous, ethanol and methanol extracts were named as GaAE, GaEE and GaME respectively while ginger aqueous, ethanol and methanol extracts were named as GiAE, Gi EE and GiME respectively. The controls methanol, ethanol and water were treated in similar fashion as described for extract preparation and checked for antimicrobial activity.

### Culture preparation

The bacterial strains were inoculated in 1 ml LB broth and grown overnight at 37°C separately before performing antimicrobial assay. The 50 μl of overnight culture of each bacterial strain was transferred separately into 5 ml of LB broth (pH 7.2) under sterile conditions and placed in shaking water bath at 37°C for 16 hours. The bacterial cells were harvested at 3000 rpm for 15 minutes at 4°C, washed twice with phosphate buffer saline (pH 7.4) and resuspended in LB broth. The inoculum concentration was adjusted to 10^7^ CFU/ml.

### Antimicrobial assay using Disc diffusion method

The antimicrobial assay of spices was performed by disc diffusion method as described by Kirby-Bauer [17]. All the experiments were performed under sterile conditions. The nutrient agar plates were inoculated separately with 10^7^ CFU of each test bacterial strain culture and evenly spread on entire surface of each plate. The sterile discs (5 mm diameter) were dipped aseptically in different extracts for one minute and placed over nutrient agar plates seeded with bacterial culture. The plates were left at ambient temperature for 15 minutes and then incubated at 37°C for 16 hours and observed for zone of inhibition. The diameter of inhibition zones was measured in milimeters. Antimicrobial assay was performed in triplicate with each bacterial strain.

### Determination of minimum inhibitory concentration (MIC)

MIC of different garlic and ginger extracts was determined by the method described by Natta et al [18] after minor modifications. The extracts were diluted ranging from 100 mg/ml to 0.01 mg/ml and checked for MIC against bacterial strains. Sterile discs were dipped in different dilutions of aqueous, ethanol and methanol extracts of garlic and ginger and placed over LB agar plates seeded with 10^7^ CFU of each bacterial cultures separately. Plates were placed at 37°C for 16 hours. The zone of inhibition in each case was measured as the diameter of the clearing zones and results were recorded. Each experiment was performed in triplicate.

### Statistical analysis

Values are mean of ± SD (standard deviation) of three replicates.

## Results and Discussion

Use of garlic and ginger as a natural supplement is considered healthy choice for the treatment of cardiovascular diseases [19,20], hypertension [21], diabetes [22]. Alzheimer's disease [23,24], inflammation, thrombosis [25] and even for cancer [26]. Recently ginger was also reported for treatment of nonalcoholic fatty liver diseases, [27]. With the increasing awareness of population toward natural therapies, spices can be considered as obvious alternate medication [28].

In the present study antibacterial effect of garlic and ginger was evaluated by disc diffusion method. The results indicated that different extracts of spices have broad spectrum antibacterial activity with variable degree of sensitivity of tested bacterial species toward these extracts. The controls did not show any antimicrobial activity. The data presented in Table 1 shows that garlic aqueous extract exhibited highest antibacterial activity against all tested bacteria except *E. coli* and *Shigella*. Garlic methanol extract was least effective against all tested bacteria. The antimicrobial activity shown by garlic extracts in this study agrees with the findings of others [13,14,29-31].

**Table 1:** Antibacterial activity of spices extracts measured as diameter (mm) of zone of inhibition

	**Garlic**			**Ginger**		
	**Aqueous Extract**	**Ethanol Extract**	**Methano Extract**	**Aqueous Extract**	**Ethanol Extract**	**Methanol Extract**
*E.coli*	14.3 ± 0.54	11.6 ± 0.27	12 ± 0	12.3 ± 0.27	15 ± 0.47	14.5 ± 0.27
*P. aeruginos*	18.3 ± 0.72	13.3 ± 0.27	11 ± 0	13 ± 0.47	14 ± 0.94	13.6 ± 0.54
*B. subtilis*	18.6 ± 0.27	13.3 ± 0.54	12 ± 0	12.3 ± 0.27	13.6 ± 0.27	11.3 ± 0.27
*Shigella*	13 ± 0.47	13.3 ± 0.54	11 ± 0	11.6 ± 0.27	15 ± 0.47	15 ± 0.47
*S. aureus*	19.3 ± 1.08	12.6 ± 0.27	11 ± 0	13 ± 0.47	13 ± 0	14.3 ± 0.27
*K. pneumoniae*	15.6 ± 0.54	14 ± 0.47	11 ± 0	11 ± 0	11 ± 0	12 ± 0.81
*S. epidermidis*	22 ± 0.47	11.6 ± 0.27	11 ± 0	12.6 ± 0.27	15 ± 0.47	12 ± 0
*S.typhi*	15.6 ± 0.56	11 ± 0	11 ± 0	11 ± 0	11.3 ± 0.27	11.7 ± 0.32

The results given in Table 1 show that ginger methanol and ethanol extracts are more effective against all tested bacterial strains than ginger aqueous extracts. *E. coli* and *Shigella* were also more susceptible to the ginger extracts. *E. coli* showed maximum susceptibility to the ginger ethanol extracts while *Shigella* showed maximum susceptibility to both ginger methanol and ethanol extract. The results of antimicrobial effect of ginger in the study are in accordance with most of the reports published regarding ginger antimicrobial activity [32-36]. The antibacterial activities of the extracts are expected perhaps due to the compounds like flavonoids and volatile oil which were dissolved in organic solvents. It is reported that sesquiterpenoids are the main component of ginger which attributes its antibacterial activity [35]. The results obtained in our study corroborate with the report of Roy et al [37], which explains that bioactive compounds of ginger rendering antimicrobial activity are volatile in nature and antimicrobial activity of ginger extract decreases upon storage. In addition to water, methanol and ethanol were also used for extract preparation as de Boer et al [38] has reported that bioactive compounds show better solubility in water miscible organic solvents.

The order of antibacterial activity of different garlic and ginger extracts against tested clinical isolates of pathogenic bacteria was as follow: 1) *E. coli, GiEE> GiME> GaAE> GiAE> GaME> GaE;* 2) *P. aeruginosa, GaAE> GiEE> GiME> GaEE> GiAE>GaME;* 3) *Bacillus subtilis, GaAE> GiEE> GaEE> GiAE>GaME> GiME;* 4) *Shigella, GiEE, GiME> GaEE> aAE> GiAE> GaME;* 5) *S.aureus, GaAE> GiME> GiEE, GiAE> GaEE> GaME;* 6) *K. pneumoniae, GaAE> GaEE> GiME> GiEE, GiAE, GaME;* 7) *S.epidermidis, GaAE> GiEE> GiAE> GiME> GaEE> GaME;* 8) *S.typhi, GaAE> GiME> GiEE> GiAE> GaEE> GaME.*

**Figure 1:**
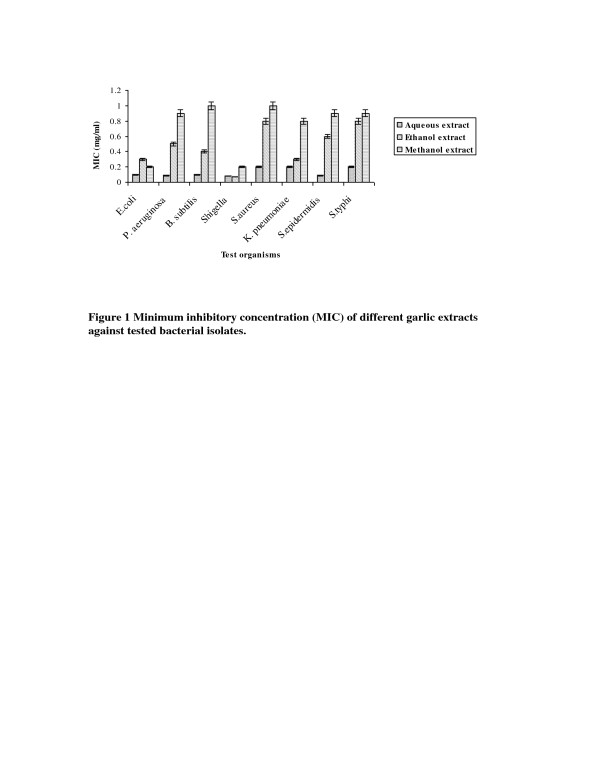
Minimum inhibitory concentration (MIC) of different garlic extracts against tested bacterial isolates.

**Figure 2:**
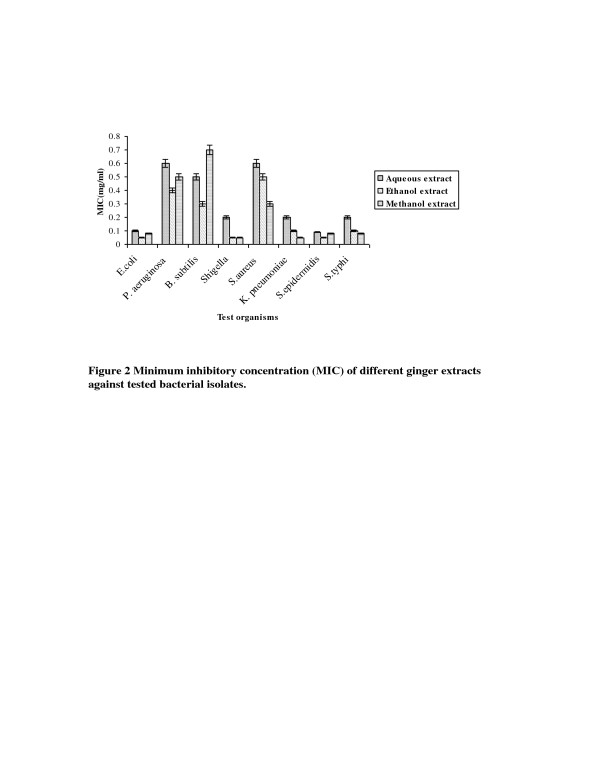
Minimum inhibitory concentration (MIC) of different ginger extracts against tested bacterial isolates.

The minimum inhibitory concentration (MIC) was determined by making the dilutions of different extracts of garlic and ginger ranging from 100 mg/ml to 0.01 mg/ml. The MIC values of different garlic and ginger extracts are summarized in Figure1 and 2 respectively. The results showed that MIC of different extracts of garlic and ginger against bacterial strains ranged from 0.05 mg/ml to 1.0 mg/ml. The data in Figure 1 indicated that all tested strains were susceptible to garlic aqueous, methanol and ethanol extract but most effective was garlic aqueous extract. From all MIC values of different garlic extracts, lowest MIC values for *E. coli, P. aeruginosa, B. subtilis, S. aureus, K. pneumoniae, S. epidermidis* and *S. typhi* were 0.1 mg/ml, 0.09 mg/ml, 0.1 mg/ml, 0.2 mg/ml, 0.2 mg/ml, 0.09 mg/ml and 0.02 mg/ml respectively with garlic aqueous extract except *Shigella* which showed lowest MIC value (0.07 mg/ml) with garlic methanol extract. Ethanol and methanol extract of ginger had lower MIC in comparison to the ginger aqueous extract against tested bacterial strains (Figure 2). In case of different ginger extracts, the lowest MIC value for *E. coli* (0.08 mg/ml), *P. aeruginosa* (0.4 mg/ml), *B. subtilis* (0.3 mg/ml) and *S. epidermidis* (0.05 mg/ml) was observed with ginger methanol extract while for *S. aureus* (0.3 mg/ml), *K. pneumoniae* (0.05 mg/ml) and *S. typhi* (0.08 mg/ml) was observed with ginger ethanol extract. The ginger methanol and ethanol extract showed lowest and same MIC value for *Shigella* (0.05 mg/ml).

The decreasing susceptibility of tested pathogenic bacteria was observed in this order: *S.epidermidis>S.aureus>B. subtilis>P. aeruginosa>K. pneumoniae = S.typhi>E. coli = Shigella.* It was interesting to note that clinical isolates, both Gram negative and Gram positive bacteria were sensitive to all tested extracts of garlic and ginger but Gram positive bacteria were more sensitive than Gram negative bacteria. This result is in accordance with the findings of Chandarana [39]; Onyeagba [40] and de-Souza [41].

It is established in the study that spices reduce and inhibit the growth of food pathogens therefore the use of spices would decrease the chances of food poisoning and increase the food shelf life. Several socioeconomic factors are major cause of miserable health condition of poor people of Pakistan which includes; poverty, unhygienic conditions, overcrowding, contamination of food /water by poor sanitary practices, limited awareness of seriousness of foodborne diseases and importance of hygiene. While living in such conditions, use of spices (garlic/ginger) in diet can reduce the risk of food contamination, protect the consumer from different foodborne diseases, improve their health status and combat with the foodborne diseases by using small quantity of spices (garlic/ginger) in diet. In this study heat effect on antimicrobial activity of garlic and ginger was not checked as it is already reported that antimicrobial activity of garlic is affected by heating at 100°C for 30–60 minutes [42]. Therefore, it is recommended to use garlic and ginger in different raw forms like pickle, garlic/ginger bread, curry powder, sauces, raw juices and without extensive cooking.

In conclusion, the results of present study have provided the justification for therapeutic potential of spices. The practice of using spices as supplementary or alternative medicine in developing countries like Pakistan will not reduce only the clinical burden of drug resistance development but also the side effects and cost of the treatment with allopathic medicine. Further clinical evaluation of spices in *in vivo* experiments is required to be carried for low cost treatment with few side effects and for prevention of recurrent infection.

## Competing interest

The authors declare that they have no competing interests.

## Authors’ contribution

All authors equally participated in designing experiments, acquisition, analysis and interpretation of data. Prof. Amin Athar critical revise the manuscript and approved the final version of manuscript. All authors read and approved the final manuscript.
